# Growth of HIV-uninfected children born to HIV-infected mothers in Guangdong, China: an 18-month longitudinal follow-up study

**DOI:** 10.1186/s12887-019-1771-5

**Published:** 2019-10-23

**Authors:** Bing Li, Liu-ying Tang, Zhi-qiang Wang, Shuang Gao, Yun-tao Wu, Hao-li Xu, Yuan-zhu Ma

**Affiliations:** Department of Healthcare, Guangdong Women and Children Hospital, No. 521, XingNan Road, Guangzhou, 511442 Guangdong China

**Keywords:** Growth in children, HIV-exposed uninfected children, HIV-infected mothers, Longitudinal follow-up

## Abstract

**Background:**

To evaluate the early growth (weight and length) of HIV-exposed uninfected (HEU) children from the prevention of mother-to-child transmission (PMTCT) of human immunodeficiency virus (HIV) program in Guangdong Province, China.

**Methods:**

A total of 731 HEU children were longitudinally followed up at 7 time points, with anthropometric measurement conducted of weight and length (supine) in the first 18 months. Z scores were calculated, with and without adjustment for gestational age.

**Results:**

A total of 708 HEU children were included in the final follow-up cohort, and 105 (14.83%) children completed all 7 follow-up visits. The mean of adjusted weight-for-age Z scores in these children was above zero and showed a decreasing trend in 18 months. The mean of adjusted length-for-age Z scores showed a decreasing trend and was above zero in the first 12 months; this declined to under zero at age 18 months. The proportion of underweight was 0.28–2.19% and that of stunting was 0.71–4.63% at each follow-up month-age. Slower growth in HEU children was associated with no sustained food subside after 6 month, mothers’ hemoglobin content less than 100 g/L during pregnancy, preterm birth, and low birth weight (*p* < 0.05).

**Conclusions:**

HEU children could catch up to WHO growth standards in first 18 months in Guangdong; however, growth declined after 12 months, and these children need sustained nutritional support.

## Background

With increased access to antiretroviral drugs (ARVs) for prevention of mother-to-child transmission (PMTCT) of human immunodeficiency virus (HIV) in China, most children born to HIV-infected mothers are not themselves infected with HIV [1, 2]. Many previous reports have shown that these HIV-exposed uninfected (HEU) children are at increased risk of mortality and have immune, growth, development, and health deficits compared with HIV-unexposed uninfected (HUU) children [3–7]. These risks in HEU children are associated with many factors, such as the health and education status of HIV-infected mothers, socioeconomic status of the family, social support, health and feeding consultation and support, and psychology intervention [8, 9]. However, many reports also have indicated that growth and development are not affected in HEU children [10–13]. Therefore, as a public health issue, the growth and health care of HEU children need to be further studied under different settings.

Since 2011 in Guangdong Province of China, free ARV prophylaxis for PMTCT has been routinely provided to all HIV-infected pregnant women from 14 gestational weeks to postpartum, as well as to their newborns in the first 4–6 weeks after birth. The PMTCT program has effectively reduced the HIV mother-to-child transmission rate to less than 5% in Guangdong [2]. However, evaluation of the growth status of these HEU children is seldom reported. A total of 731 HEU children were longitudinally followed up from birth to age 18 months in this study. The children’s weight and length were measured and analyzed to evaluate the early growth of HEU children from the program of PMTCT of HIV in Guangdong, and to provide scientific evidence for the health care of these children.

## Methods

### Study design and participants

The present longitudinal cohort study evaluating the growth of HIV-uninfected children born to HIV-infected mothers (called HEU children in this study) was conducted in Guangdong Province, China from January 2012 to December 2016. A total of 731 HEU children included in the free HIV PMTCT program were recruited at birth in the hospital from January 2012 to June 2015. After written informed consent was obtained from parents or guardians, the children were followed up from birth to age 18 months, with anthropometric measures taken of weight and length (supine). We excluded the data of HEU children who were twins or multiple births, those who were HIV-infected, and those who died within the first 18 months. Finally, 708 eligible children were included in the original cohort and 105 children were followed up at all 7 time points.

### Follow-up

HEU children were followed up by local child health care doctors and nurses at birth in a delivery hospital, and they were followed up in a child health care service hospital at ages 1, 3, 6, 9, 12, and 18 months. Information of social demographic status, prenatal complications (included pregnancy with internal or surgical diseases, amniotic fluid abnormality, placenta and umbilical cord abnormality, preterm, fetal intrauterine abnormality, low birth weight, postpartum hemorrhage, puerperal infection et al.), CD4+ T cell level, and ARVs use by the mother during pregnancy were derived from the perinatal medical records and routine case report cards during the period of delivery in the hospital. At each time point of monthly planned visits, children underwent follow-up visits, including measurement of weight and length (supine) using routine calibrated instruments, physical examination, feeding consultation, and recording of the feeding status or any hospitalization for illness in children during the interval since the last follow-up visit. At the same time, children were referred for free services of the government PMTCT program, including HIV testing, provision of ARVs (azidothymidine or nevirapine) for HIV PMTCT in the child’s first 4 to 6 weeks of life, and provision of 3000 RMB to purchase infant formula milk in the first 6 months after birth.

### Confirmation of HIV infection status of children

The HIV status of children was confirmed either by western blot analysis after seropositive tests results for HIV antibodies at ages 12 and 18 months or by HIV DNA polymerase chain reaction (PCR) tests using dried blood spot (DBS) specimens at ages 6 weeks to 3 months. Western blot analysis was conducted at the local city-level laboratory of the Chinese Center for Disease Control and Prevention using venous blood specimens; DBS testing was conducted in the Administrative Authorized HIV-testing laboratory of Guangdong Province Hospital for Women and Children Healthcare.

### Statistical analysis

Statistical analysis was conducted using R software version 3.5.1 (2018-07-02). We compared the difference of characteristics between all 708 children who were ever followed up and 105 children who were followed up at all 7 time points by a chi-square test. According to the World Health Organization (WHO) Child Growth Standards in 2006 [14], we calculated the anthropometric Z scores of weight and length for each child by the formula Z = (X - median)/SD (X was anthropometric measures numerical value of weight and length of children, median was the WHO standard value of median of weight and length, SD was the WHO standard value of standard deviation of weight and length of children), both with and without adjusting for gestational age at delivery. As to preterm HEU children, if the adjusted month-age was less than zero, weight and length were adjusted using the reference gestational age of Indonesia [15].

Underweight was defined as weight-for-age Z scores less than − 2, overweight as weight-for-age Z scores more than 2, and stunting as length-for-age Z scores less than − 2. The mean and 95% confidence interval (CI) and median of Z scores of weight for age, gestational age-adjusted weight for age, length for age, and gestational age-adjusted length for age were calculated and trend lines were plotted. Generalized linear mixed models (GLMM) were used to evaluate the effect of covariates on growth over time, and the age month was assigned to be a group variable, and other characteristics variables was assigned to be the covariates. Results were considered statistically significant with *p*-value < 0.05.

## Results

### Flow of follow-up among children

**Fig. 1:**
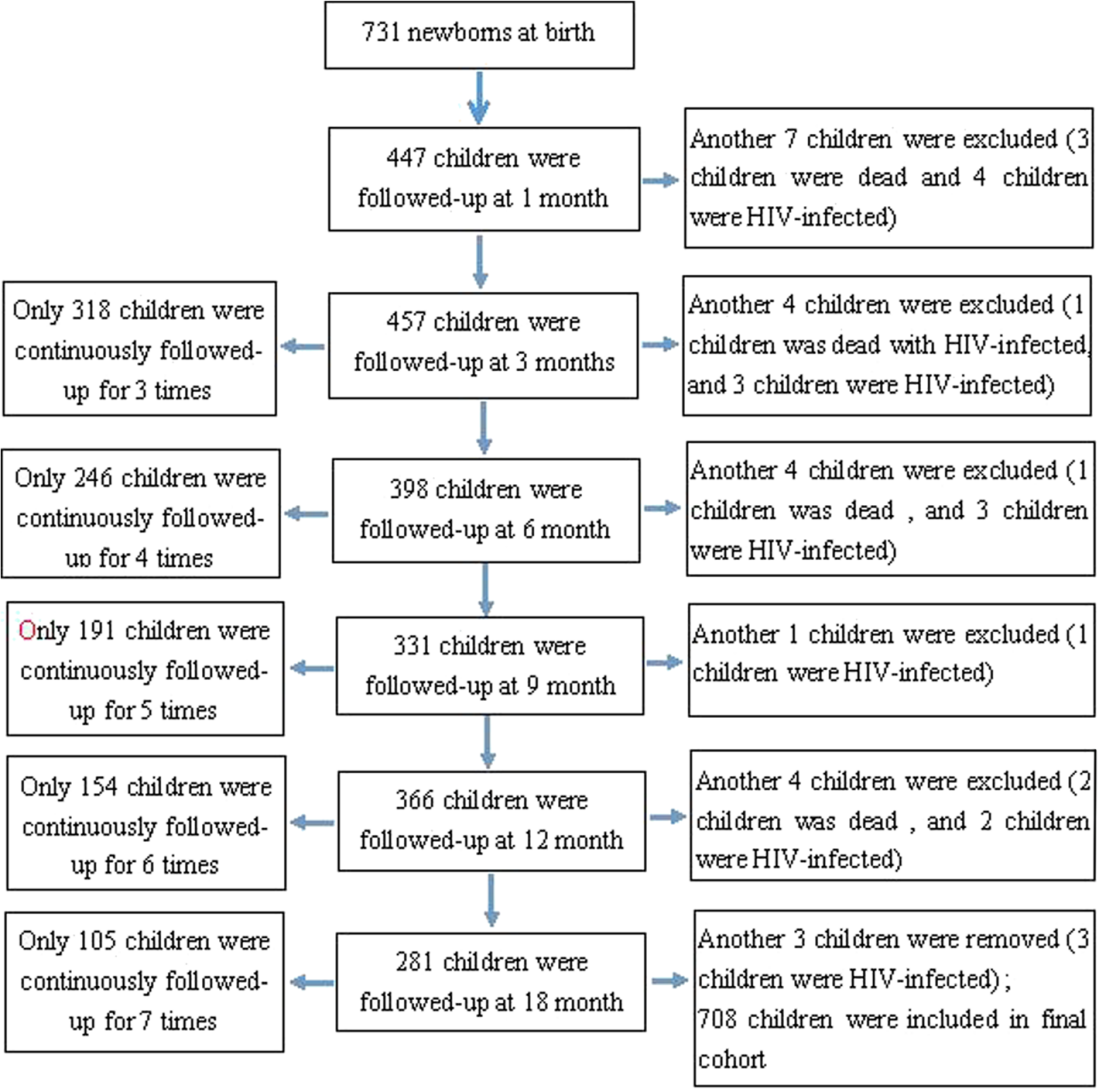
Flow of the follow-up cohort of 731 children born to HIV-infected mothers

A total of 731 newborns born to HIV-infected mothers were included in the original follow-up cohort at birth. During the follow-up period of 18 months, 23 children were excluded from the original cohort because 6 children died, 16 children were HIV-infected, and 1 child died as a result of HIV infection. Finally, 708 HEU children were included in the cohort. Among 708 HEU children, 105 (14.83%) children underwent all 7 follow-up visits (including the first visit at birth); 133 (18.79%) children completed 6 visits; 102 (14.41%) children attended 5 visits; 87 (12.29%) children underwent 4 visits; 96 (13.56%) children attended 3 visits; 124 (17.51%) children completed 2 visits; and 61 (8.62%) children underwent 1 follow-up visit. The flow of the follow-up cohort of children is shown in Fig. 1.

### Characteristics of children and their HIV-infected mothers

Characteristics of children and their HIV-infected mothers are shown in Table 1. In all invested characteristics, only infants who were formula fed in the first 3 months and in the first 6 months showed significant differences between 708 ever followed-up children and the 105 seven-time followed-up children. In the 105 seven-time followed-up children, 94.29% (99/105) were formula fed in both the first 3 months and in the first 6 months; however, the respective proportions were only 41.10% (=36.24, *P* < 0.001) and 32.49% (= 47.31, *P* < 0.001) of all 708 children.

**Table 1:** Characteristics of 708 ever followed-up children and their HIV-infected mothers

Characteristics of subjects	n	%
Mothers’ age		
<25 years	170	24.01
≥25 years and < 35 years	442	62.43
≥35 years	96	13.56
Mothers’Educational leve		
Primary school and below	112	15.82
Junior high school	401	56.64
Senior high school	120	16.95
College or university	43	6.07
Unknown	32	4.52
Currentdwelling place		
Local city of registered residence	587	82.91
Other citiesout of registered residence	121	17.09
Childbearing history of mothers		
Primipara	285	40.25
Pluripara	423	59.75
Mothers’ hemoglobin content^a^		
<100 g/L	179	25.28
≥100 g/L	529	74.72
Newborn preterm birth		
Yes	74	10.45
No	634	89.55
Perinatal complications^b^		
Yes	112	15.82
No	596	84.18
Mothers used ARVs^a^		
Yes	498	70.34
No	210	29.66
CD4^+^ level of mothers^a^		
≥500/mm^3^	60	8.47
≥350/mm^3^and < 500/mm^3^	41	5.79
≥200/mm^3^and < 350/mm^3^	44	6.21
<200/mm^3^	43	6.07
Unknown	520	73.45
Children gender		
Female	306	43.22
Male	402	56.78
Children low birth weight		
Yes	98	13.84
No	610	86.16
Children hospitalization		
Yes	36	5.08
No	672	94.92
Infants feeding in 3 months		
Formula feeding	291	63.68
Other feeding ^c^	166	36.32
Infants feeding in 6 months		
Formula feeding	230	57.79
Other feeding ^c^	168	42.21
Newborns used ARVs^d^		
Yes	622	87.85
No	86	12.15

^a^These characteristics were the status during pregnancy

^b^Perinatal complicationsdidn’t included anemia and preterm

^c^Other feeding included exclusive breast-feeding and others food mixed artificial feeding, there were only 9 children was exclusive breast-feeding in 3 month and only 1 child was exclusive breast-feeding in 6 month

^d^*ARVs* antiretroviral drugs

### Weight of children during follow up

**Fig. 2:**
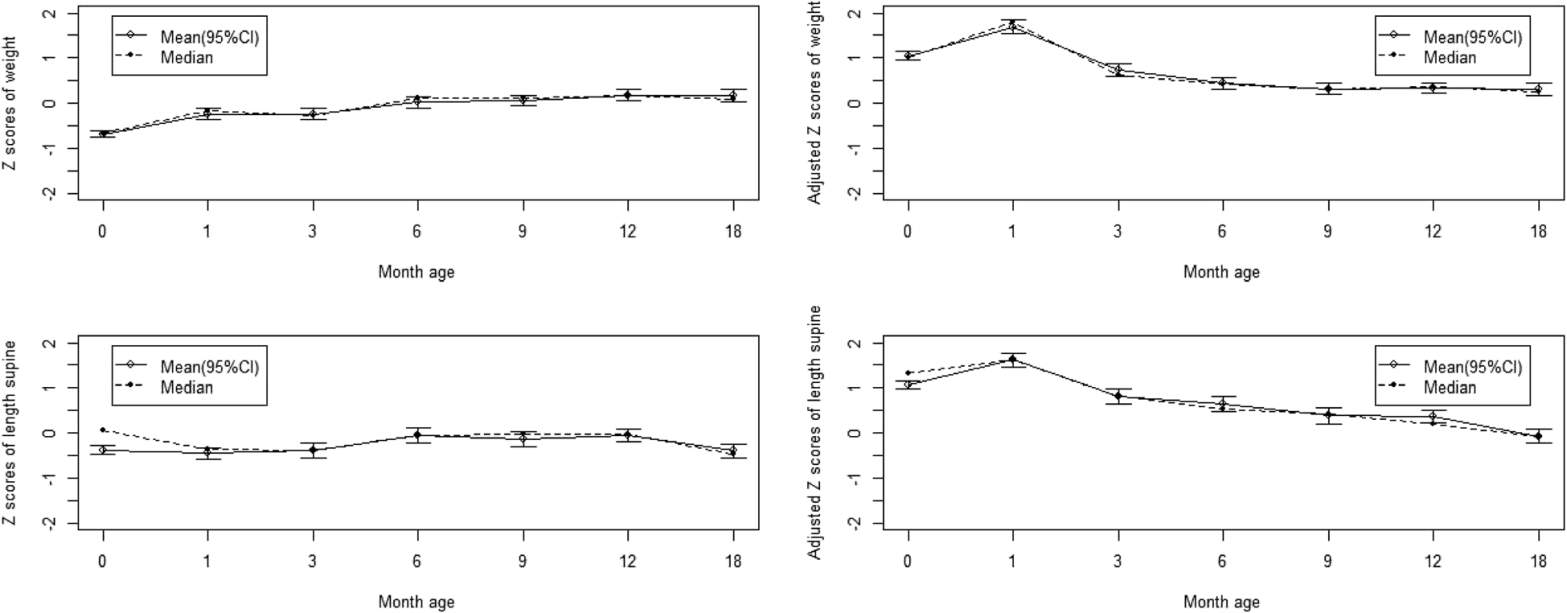
The Z scores of gestational-age-adjusted weight and length for age among all 708 ever followed-up HEU children

The trend of the mean and 95% CI and median of weight-for-age Z scores and that of gestational-age-adjusted weight-for-age Z scores of HEU children are shown in Figs. 2. In 708 ever followed-up children, the unadjusted Z score mean of weight for age in both groups showed an increasing trend from less than − 0.5 to near zero. However, after adjusting for gestational age, all adjusted Z score means of weight-for-age were above zero and showed a decreasing trend in the first 18 months, with a higher level in the first 3 months. And that tendency was same in the 105 seven-time followed-up children.

The gestational age-adjusted weight-for-age Z scores of 105 seven-time followed-up HEU children who had characteristics with significant differences (*p* < 0.05) are shown in Table 2. HEU children whose mothers’ hemoglobin content less than 100 g/L during pregnancy, children with preterm, had lower adjusted weight-for-age Z scores at 1 month and at 3 month.

**Table 2:** The Z scores of gestational age-adjusted weight/length for age in 105 different characteristic seven-time followed-up HEU children

Characteristic	n	At birth		1th month		3th month		6th month		9th month		12th month		18th month	
		Mean ± Sd	*P* value	Mean ± Sd	*P* value	Mean ± Sd	*P* value	Mean ± Sd	*P* value	Mean ± Sd	*P* value	Mean ± Sd	*P* value	Mean ± Sd	*P* value
scores of GAAW															
Mothers’ hemoglobin content^a^															
<100g/L	29	0.48 ± 1.07	*P =* 0.004	1.30 ± 1.73	*P =* 0.001	0.78 ± 1.60	*P =* 0.999	0.27 ± 1.10	*P =* 0.438	0.11 ± 1.05	*P =* 0.415	0.33 ± 1.14	*P =* 0.925	0.64 ± 1.66	*P =* 0.284
≥100g/L	76	1.25 ± 1.18		2.13 ± 1.60		0.75 ± 1.21		0.46 ± 0.93		0.33 ± 1.07		0.38 ± 1.12		0.36 ± 1.16	
Newborn preterm birth															
No	93	1.01 ± 1.20	*P =* 0.379	1.75 ± 1.54	*P =* 0.000	0.63 ± 1.26	*P =* 0.004	0.36 ± 0.98	*P =* 0.278	0.27 ± 1.02	*P =* 0.926	0.43 ± 1.11	*P =* 0.134	0.47 ± 1.34	*P =* 0.483
Yes	12	1.29 ± 1.18		3.10 ± 2.18		1.73 ± 1.42		0.76 ± 0.91		0.29 ± 1.39		-0.14 ± 1.11		0.22 ± 1.07	
Z scores of GAAL															
Children low birth weight															
No	94	1.21 ± 1.29	*P =* 0.002	1.86 ± 1.98	*P =* 0.228	0.91 ± 1.26	*P =* 0.003	0.95 ± 1.68	*P =* 0.563	0.25 ± 1.65	*P =* 0.846	0.25 ± 1.13	*P =* 0.348	-0.12 ± 1.15	*P =* 0.142
														-0.84 ± 1.04	

^a^.Mothers’ hemoglobin contentwas the status during pregnancy

*HEU* HIV-exposed uninfected

*GAAW* gestational age-adjusted weight

*GAAL* gestational age-adjusted length

The Z scores of weight for age among the seven-time followed-up children compared with that among less than seven-time follow-up children, at birth was (*n* = 105, 1.04 ± 1.20 vs. *n* = 603, 1.05 ± 1.22; t = − 0.07, *P =* 0.945), at 1 month was (*n =* 105, 1.90 *±* 1.67 vs. *n =* 342, 1.63 *±* 1.72; *t =* 1.84, *P =* 0.066), at 3 month was (*n =* 105, 0.76 *±* 1.32 vs. *n =* 352, 0.70 *±* 1.49; *t =* 0.38, *P =* 0.702), at 6 month was (*n =* 105, 0.41 *±* 0.98 vs. *n =* 293, 0.45 *±* 1.27; *t =* − 0.26, *P =* 0.797), at 9 month was (*n =* 105, 0.27 *±* 1.06 vs. *n =* 226, 0.34 *±* 1.17; *t =* − 0.44, *P =* 0.663), at 12 month was (*n =* 105, 0.36 *±* 1.12 vs. *n =* 261, 0.34 *±* 1.15; *t =* 0.18, *P =* 0.854), and at 18 month was (*n =* 105, 0.44 *±* 1.31 vs. *n =* 176, 0.24 *±* 1.20; *t =* 1.23, *P =* 0.218).

In all 708 HEU children, at birth, 1, 3, 6, 9, 12, and 18 months, the proportion of underweight (gestational age-adjusted weight-for-age Z scores less than − 2) was 0.28% (2/708), 0.67% (3/447), 2.19% (10/457), 2.01% (8/398), 1.51% (5/331), 0.82% (3/366), and 1.78% (5/281); and that of overweight (gestational age-adjusted weight-for-age Z scores more than 2) was 20.90% (148/708), 40.72% (182/447), 19.91% (91/457), 8.29% (33/398), 7.55% (25/331),8.20% (30/366), and 6.05% (17/281), respectively. In addition, there was a higher proportion of underweight after age 1 month.

### Length (supine) of followed-up children

The trend of the mean and 95% CI and median of length-for-age Z scores and that of gestational age-adjusted length-for-age Z scores of HEU children are shown in Figs. 2. In the 708 ever followed up, mean length-for-age Z scores were less than zero and showed an increasing trend in the first 6 months but a decreasing trend from age 6 to 18 months; After adjusting for gestational age, the adjusted Z score means of length for age were above zero in the first 12 months and showed a decreasing trend, which declined to less than zero at 18 months. And that tendency was same in the 105 seven-time followed-up children.

The gestational age-adjusted length-for-age Z scores in 105 seven-time followed-up HEU children with characteristics that showed significant differences (*p* < 0.05) are shown in Table 2. HEU children with low birth weight had lower adjusted length-for-age Z scores only at birth.

The Z scores of length for age among the seven-time followed-up children compared with that among less than seven-time follow-up children, at birth was (*n =* 105, 1.05 *±* 1.41 vs. *n =* 603, 1.07 *±* 1.03; *t =* − 0.17, *P =* 0.866), at 1 month was (*n =* 105, 1.79 *±* 1.92 vs. *n =* 342, 1.57 *±* 1.71; *t =* 1.28, *P =* 0.200), at 3 month was (*n =* 105, 1.07 *±* 2.07 vs. *n =* 352, 0.74 *±* 1.98; *t =* 1.94, *P =* 0.053), at 6 month was (*n =* 105, 0.92 *±* 1.65 vs. *n =* 293, 0.54 *±* 1.75; *t =* 2.11, *P =* 0.035), at 9 month was (*n =* 105, 0.23 *±* 1.61 vs. *n =* 226, 0.45 *±* 1.56; *t =* − 1.21, *P =* 0.226), at 12 month was (*n =* 105, 0.20 *±* 1.12 vs. *n =* 261, 0.44 *±* 1.38; *t =* − 1.35, *P =* 0.177), and at 18 month was (*n =* 105, − 0.19 *±* 1.15 vs. *n =* 176, 0.00 *±* 1.34; *t =* − 0.99, *P =* 0.324).

In all 708 HEU children, at birth, 1, 3, 6, 9, 12, and 18 months, the proportion of stunting was 0.71%(5/708), 1.57%(7/447), 4.60%(21/457), 4.02%(16/398), 4.53%(15/331), 2.73%(10/366), and 4.63%(13/281) respectively.

## Discussion

A previous review report has shown that HIV prevalence among pregnant women in China who access antenatal care remains below 0.1% [1]. The HIV vertical transmission rate has substantially decreased to less than 5% [1, 2]. However, with more than 17 million children born each year in China, growth among HEU children should be considered a public health issue. In this study, we found that the mean unadjusted weight-for-age and length-for-age Z scores showed increasing trends to nearly zero in the first 6 months among HEU children. However, these became decreasing trends above zero after adjusting for gestational age, in addition, there was only 10% of the total cohort HEU children were preterm, and there was not a food subsidy after 6 month, and there were no significant differences of adjusted weight-for-age and length-for-age Z scores between 7 follow-up visits children and less than 7 follow-up visits children. We think that preterm birth might mainly influence the growth of HEU children in the first 3 months, but sustained nutritional support over 18 months was the major factor influencing growth in these HEU children. We did not find an obviously higher rate of preterm birth among HEU children compared with previously reported rates of the general population [16, 17]. We also showed that both adjusted and unadjusted weight-for-age Z scores were slightly above zero from age 6 to 18 months, but that both adjusted and unadjusted length-for-age Z scores showed a decreasing trend to less than zero at age 18 months, which suggests that health interventions among HEU children in Guangdong Province are effective for weight but not length, or that HIV exposure might have a greater impact on length than on weight in HEU children. Previous studies have also shown that HEU children are at increased risk of stunting [18] or have poorer early growth [19], compared with their HUU peers. Another study showed that HEU infants have poorer growth than HIV-unexposed infants in the first 12 months of life [20]; however, that study showed that growth in HEU children was poorer after 6 months compared with the first 6 months, which might be because HEU children had access to funding assistance to purchase formula milk in the first 6 months. In our study, we also find that there was a high rate of overweight at each age of follow-up, a reminder that overweight among HEU children is a concern in Guangdong. A study in the United States also reported that 13% of HEU children are obese, which might be related to maternal use of Tenofovir [21]; another study did not find an association between meconium Tenofovir concentration and infant growth [22]. A study from Brazil reported that in HEU children, early exposure to ARVs was associated with lower growth up to 2 years of age [23]. Yet that study did not find an association between maternal or newborn ARV use and growth in HEU children.

Feeding is one of main factors influencing the growth of infants. In China, formula feeding consultation and a subsidy of 3000 RMB for formula milk are routinely provided for all children born to HIV-infected mothers. Our results showed that there was a significantly higher proportion of formula-fed infants at 3 months (94.29% vs. 63.68%) and 6 months (94.29% vs. 57.79%) among the 105 HEU children followed up at all 7 time points compared with the 708 ever followed-up HEU children. However, there were no differences in growth between the different feeding models in both two groups of children. In this study, few HEU children were exclusively breast fed. These results might indicate that artificial feeding, including formula feeding for HEU infants in Guangdong in the first 6 months is suitable for their growth of weight and length; however, feeding consultation and support for these children should be reinforced after 6 months. Previous studies have found that early growth in HEU children is lower than that in HUU children, partly owing to mastitis with breastfeeding that cannot be resolved with intensive support for exclusive breastfeeding [24]. Interventions are needed during weaning of all HEU breast-fed children [18]. A study in India showed that formula-fed infants born to HIV-infected mothers experienced severe malnutrition during the first 2 months of life, and that study included HIV-infected infants [25]. A study in Uganda reported that the increased risk of morbidity among HEU children aged 6–11 years can be partially explained by early cessation of breastfeeding [26]. One study has shown that the benefits of breastfeeding in HEU children may related to the oligosaccharide composition of breast milk [27]. We found that formula feeding and artificial feeding could achieve good growth in HEU children in Guangdong; this result is similar to a study from Africa [28]. All these studies indicate that proper and persistent feeding consultations and support for HEU children, according to their individual condition, is important for their optimal growth. And the introduction of complementary feeds after age 6 months also should be paid more attention to.

The results of GLMM also showed that preterm and low birth weight might are both the influencing factors on growth among HEU children in the first 3 month, but a potential long-term effect of preterm and low birth weight on the growth of these children need further study as the cases were not enough in our study. We also found that mothers’ hemoglobin content less than 100 g/L during pregnancy was associated with lower gestational age-adjusted weight-for-age Z scores in the first month. These results suggest that the growth of HEU children should continue to be intervened from pregnancy throughout childhood in this population. Many previous reports have shown that the health and development problems of HEU infants are multi-factorial [8, 9], and different studies report that these factors have different impacts on growth in terms of weight and length among HEU children. Thus, maintenance of optimal growth in HEU children is dependent on integrated and specific support that is provided consistently from pregnancy throughout childhood, as reported in many previous studies [19, 29]. We found that HEU children who completed all seven follow-up visits had a lower proportion of underweight.

In this study, we used the WHO Child Growth Standards in 2006 but we used a growth reference for China to calculate Z scores and adjusted Z scores of weight for age and length for age. Previous studies have shown that the WHO growth standards might be more conservative in estimating undernutrition and are more applicable for international comparisons with Chinese children [30]. There is no openly reported growth standard of birth weight and length for preterm for gestational age in China or of the WHO; therefore, we used the standard for Indonesia to calculate adjusted Z scores of birth weight and length in this study, as both Guangdong Province in China and Indonesia are located in southeast Asia.

One limitation of this study is the large loss to follow-up over the seven time points, which was owing to a change in the method of contact and patient relocation. But we found no significant differences between the group of ever followed-up children and the group who completed all seven follow-up visits, except for infant formula feeding in the first 3 months and the first 6 months, which could mitigate the biases of loss to follow-up. Another limitation of this study was that we had no control follow-up group; however, the purpose of this study was to evaluate the growth status of weight and length among HEU children, which can be accomplished by comparison with the WHO growth standards. And our results also showed that HEU children had a similar growth pattern as general children in China, and the pattern is the decreasing prevalence of low weight and growth stunt and increasing prevalence of overweight and obesity of children [31, 32].

## Conclusion

In conclusion, HEU children from the PMTCT of HIV program could catch up to WHO growth standards in first 18 months in Guangdong; however, growth declined after 12 months, and these children need sustained nutritional support. Growth in terms of weight and length in HEU children is associated with, mothers’ hemoglobin content less than 100 g/L during pregnancy, preterm birth, and low birth weight. Findings also highlight the necessary to provide continued professional support and intervention to improve growth in HEU children, from pregnancy throughout their entire childhood.

## Data Availability

The dataset used and analyzed in the current study is available from the corresponding author on reasonable request.

## Abbreviations

ARVs: antiretroviral drugs
CI: confident interval
GLMM: Generalized linear mixed models
HEU: HIV-exposed uninfected
HIV: human immunodeficiency virus
HUU: HIV-unexposed uninfected
PMTCT: prevention of mother-to-child transmission
